# Self-Reported COVID-19 Vaccine Status and Barriers for Pediatric Emergency Patients and Caregivers

**DOI:** 10.5811/westjem.18417

**Published:** 2024-10-29

**Authors:** Amanda M. Szarzanowicz, Kendra Fabian, Maya Alexandri, Carly A. Robinson, Sonia Singh, Michael Wallace, Michelle D. Penque, Nan Nan, Changxing Ma, Bradford Z. Reynolds, Bethany W. Harvey, Heidi Suffoletto, E. Brooke Lerner

**Affiliations:** * State University of New York at Buffalo, Jacobs School of Medicine and Biomedical Sciences, Department of Emergency Medicine, Buffalo, New York; † University of Wisconsin-Madison, School of Medicine and Public Health, BerbeeWalsh Department of Emergency Medicine, Madison, Wisconsin; ‡ Medical College of Georgia at Augusta University, Department of Emergency Medicine, Augusta, Georgia; § University of California Davis School of Medicine, Department of Emergency Medicine, Sacramento, California; ∥ State University of New York at Buffalo, Jacobs School of Medicine and Biomedical Sciences, Department of Pediatrics, Buffalo, New York; ** State University of New York at Buffalo, School of Public Health and Health Professions, Department of Biostatistics, Buffalo, New York; †† State University of New York at Buffalo, Jacobs School of Medicine and Biomedical Sciences, Department of Surgery, Buffalo, New York; ‡‡ State University of New York at Buffalo, Jacobs School of Medicine and Biomedical Sciences, Department of Orthopedics, Buffalo, New York; a Posthumous author

## Abstract

**Objective:**

This study determined if the caregivers of children in the emergency department (ED) have the same COVID-19 vaccination status as the child, the reasons they chose to not vaccinate the child, and self-identified barriers to vaccination to determine if the ED is appropriate for vaccination intervention.

**Methods:**

A survey was administered to caregivers of pediatric ED patients at four Children’s Hospitals in: Augusta, GA, Buffalo, NY, Madison, WI, and Sacramento, CA. Participants were asked about their and the child’s demographics, vaccination status, and barriers to vaccination. We used descriptive statistics, Cohen's kappa, and logistic regression to analyze responses.

**Results:**

941 caregivers were considered for enrollment, and 800 consented to participation. Participants were 75% women with a mean age of 40.9 ± 8.9 years. 51% (409) of the pediatric ED patients were COVID-19 vaccinated, as were 74% (591) of the caregivers. There was variation across sites, but overall, 15% of caregivers of unvaccinated children wanted the child tobe vaccinated with the most common barriers to vaccination identified as safety data (25%), time availability (20%), and ability to obtain an appointment (13%). The most common reason for not wanting the child COVID-19 vaccinated was concern the vaccine didn’t work or had too many side effects.

**Conclusion:**

A small but clinically important group of pediatric ED patients are not COVID-19 vaccinated but their caregivers want them to be vaccinated, indicating that consideration should be given to offering vaccination in the ED. Reasons for avoiding COVID-19 vaccination were primarily concerns with efficacy and side effects.

Population Health Research Capsule What do we already know about this issue?

*Vaccination is a useful intervention in epidemics by promoting herd immunity. The ED is often used as a safety net to provide primary care for underserved populations.*

What was the research question?

*Should the COVID-19 vaccine be offered in the pediatric ED?*

What was the major finding of the study?

*At four children’s hospitals, the majority (73%) of adults accompanying children to the ED had the same vaccination status against COVID-19 as the pediatric patient. However, 15% of caregivers wanted to have their unvaccinated child vaccinated.*

How does this improve population health?

*The pediatric ED may be an adequate site for COVID-19 vaccination as a significant portion of the adult companions of unvaccinated pediatric patients desired vaccination for their child.*

## INTRODUCTION

Vaccination has been a key intervention in reducing the burden of COVID-19 on society and the healthcare system. Vaccination provides protection against COVID-19 and its short- and long-term complications, including preventing multisystem inflammatory syndrome in children after a COVID-19 infection.^1^ The COVID-19 vaccination also mitigates the risk of children acting as disease vectors for high-risk populations and reduces the burden placed on families when an infected child needs to be quarantined from childcare or school.^2^ The COVID-19 vaccine was approved for emergency use in children ages 5–17 and as of August 2022, 38% of US children aged 5–11 and 70% of children aged 12–17 have received at least one dose of a COVID-19 vaccine.^3^ In contrast, 57% of US children aged 5–12 and 48.1% of children 13–17 received the flu vaccine for the 2021–2022 season.^4^

The use of the emergency department (ED) to deliver vaccines like the COVID-19 vaccine may be a way to increase vaccination rates, especially during a pandemic when in-person primary care visits are limited. After recognizing the need for more targeted efforts to vaccinate those who had not yet sought the vaccine, or remained hesitant, and the potential role of the ED as a healthcare safety net during a pandemic, a survey was conducted of adults presenting to an ED in Buffalo, NY to identify vaccination rates among ED patients and barriers to vaccination. The authors of that study found that adult ED patients were vaccinated at a slightly lower rate than the general population and that a small but significant portion of those who were unvaccinated desired to be vaccinated, suggesting that the ED may be a suitable location to vaccinate adults against COVID-19.^5^ The study did not consider children, a population in which vaccination hesitancy has historically been heightened stemming from false claims that autism spectrum disorder may be attributed to childhood vaccinations in the 1998 paper published in *The Lancet*,^6^ which was subsequently discredited and the principal author Andrew Wakefield struck from the United Kingdom medical register. Studies have shown that providing the influenza vaccine in a pediatric ED (PED) can increase vaccination rates and overcome hesitancy.^7^ ^,^ ^8^

Vaccination rates for COVID-19 among children in the US vary greatly between states.^3^ For this reason we chose to survey four different regions of the country. Little is known about the vaccination rate of the PED population. In this study we aimed to determine whether the adult companions of children in the ED had the same vaccination status as the child they were accompanying, the reasons they chose not to vaccinate the child they were with, and any self-identified barriers to vaccination of the child.

## METHODS

We conducted a researcher-administered survey in four EDs providing pediatric emergency care in geographically diverse areas of the United States. The survey was conducted from June 15–October 28, 2022 with sites starting enrollment at different times based on when they received institutional review board (IRB) approval. The surveys were conducted when research assistants (RA) were available in the ED with the goal of collecting 200 surveys at each site. This study was approved by the IRBs at each site: Augusta University in Augusta, GA; State University of New York at Buffalo in Buffalo, NY; University of California Davis in Sacramento, CA; and University of Wisconsin Madison in Madison, WI. Each participant provided verbal consent to participate in the survey.

### Setting

The survey was conducted at four pediatric specialty hospitals in the US. In Augusta, GA, the site was the region’s only children’s hospital, which sees approximately 30,000 ED visits per year. The hospital is in Richmond County with a population just over 200,000 people. In Buffalo, NY, the site was the region’s only children’s hospital, which sees 45,000 visits per year. The hospital is in Erie County with a population of just over 950,000 people. In Madison, WI, the site was the region’s only children’s hospital, which sees 18,000 visits per year. The hospital is in Dane County with a population just under 270,000 people. In Sacramento, CA, the site is one of three children’s hospitals, which sees 10,000 visits per year. The hospital is in Sacramento County with a population just over 1.5 million people.

### Eligibility Criteria

All patients between 5–17 years of age who presented to a participating ED were considered for enrollment in the study. Triage category, chief complaint, and patient demographics were recorded from the patient’s chart. The RA then approached the patient’s clinician to determine whether the adult accompanying the patient could be approached for the survey. Reasons that the adult accompanying the patient was not able to be approached included infectious precautions for the patient, patient was too ill, patient was actively receiving medical care, the adult did not speak English, the patient was sleeping, or the clinician identified that it was not appropriate for them to be approached at that time. If the patient’s adult companion could be approached for the survey, the RA entered the room and obtained verbal consent.

### Data Collection

After consent was obtained, the survey was verbally administered to the adult companion of the patient and the answers were recorded using REDCap 10.3.3 (Research Electronic Data Capture, Vanderbilt University, Nashville, TN) (hosted at Jacobs School of Medicine and Biomedical Sciences) on an iPad (Apple Inc., Cupertino, CA). The survey questions are available in Appendix 1. Pediatric ED (PED) patient and adult companion demographic characteristics collected included age at presentation, race, ethnicity, and gender. We also collected education level, insurance type, and where the respondent reported obtaining their information on COVID-19.

Pediatric patients and adult companions were classified as COVID-19 vaccinated if they had received at least one dose of the vaccine. Those who were not vaccinated were divided into those who wanted to get the vaccine and those who did not want to get the vaccine. For queries regarding barriers to care, the question was read exactly to the participant, but answer choices were not provided. Instead, as the participant stated their response, the RA categorized the response or recorded it as “other,” and wrote down what the participant had stated. For responses that did not fit one of the given categories, RAs documented them and one author (EBL) then classified them. If the response could not be classified, it was defined as “other” for analysis.

### Data Analysis

Once data collection was completed, it was exported from REDCap and analyzed using SAS 9.4 (SAS Institute Inc, Cary, NC). We used descriptive statistics, Cohen’s kappa, and logistic regression to analyze the survey responses. We excluded cases from further analysis if data was missing. We developed two logistic regression models to assess the effects of demographic factors on the adult companion’s COVID-19 vaccination status and PED patient’s COVID-19 vaccination status separately, where adult companion and child’s COVID-19 vaccination status were both a binary response (vaccinated, not vaccinated), and different factors were used in two separate, logistic models. One logistic model was developed to assess the effects of adult clinical site on the adult companion’s COVID-19 vaccination status. We built a second logistic regression model to investigate the effects of the study site on the PED patient’s COVID-19 vaccination status. We excluded cases with missing data from these models. Finally, we used Cohen’s kappa to measure the agreement between COVID-19 vaccination status of adult companion and PED patient.

## RESULTS

Between the four study sites, 941 adult companions were considered for enrollment, and 800 (85%) were able to be approached and consented to participate (Table 1). Of the participants, 75% were women and the mean age was 40.9 ± 8.9 years. In contrast, the PED patients were 50% female. When compared across study sites, there were differences in demographics that generally aligned with the participating hospitals’ catchment areas (Table 2). Approximately half of the PED patients and three-quarters of their adult companions were vaccinated against COVID-19. There was variation across the sites, but for 15% (56) of the PED patients who were not COVID-19 vaccinated, the respondent stated that they wanted the child to be vaccinated. These 56 cases represented 7% of the total interviewed sample. Further, 92% (375) of the vaccinated PED patients and 97% (571) of the vaccinated adult companions reported receiving the recommended second COVID-19 vaccine dose or the single dose if they had received the Johnson & Johnson vaccine.

**Table 1.** Individuals considered for enrollment and enrolled in the study compared by site.

	Total	Buffalo, NY	Davis, CA	Madison, WI	Augusta, GA
Considered for enrollment	941	261	230	241	209
Staff agreed to let research assistant approach	904	249	219	230	206
Reason not approached*		4 - patient sleeping 2 - adult did not speak English 2 - patient/adult not present in room 4 - staff not asked	1 - patient sleeping 1 - adult did not speak English 1 - patient/adult not present in room 1 - adult too upset 1 - unknown 6 - staff not asked	3 - adult did not speak English 1 - patient receiving medical care 2 - adult too upset 1 - infectious precautions 4 - staff not asked	2 - patient receiving medical care
Consented to participate	800	200	200	200	200

Note: In 13 cases staff were not asked first, but patient consented (3 Buffalo, 6 Davis, 3 Madison, 1 Augusta).

**Table 2.** Description of the included subjects compared by site.

		Total N = 800	Buffalo n = 200	Davis n = 200	Madison n = 200	Augusta n = 200
Gender of participant	Male	22% (174)	22% (44)	29% (57)	22% (43)	15% (30)
	Female	75% (600)	77% (154)	71% (141)	78% (155)	75% (150)
	Other or missing	3% (26)	1% (2)	1% (2)	1% (2)	10% (20)
Gender of patient	Male	49% (394)	49% (97)	42% (83)	55% (109)	53% (105)
	Female	50% (402)	52% (103)	59% (117)	44% (88)	47% (94)
	Other or missing	1% (4)	0% (0)	0% (0)	1% (3)	<1% (1)
Age of participant	Mean years (SD)	40.9 (8.9)	40.4 (8.9)	41.3 (8.8)	41.5 (6.6)	40.2 (10.6)
Age of patient	Mean years (SD)	11.2 (5.1)	11.7 (7.4)	10.6 (4.0)	11.6 (4.0)	10.8 (4.3)
COVID-19 vaccination status participant	Received any vaccine	74% (591)	80% (159)	74% (148)	82% (163)	61% (121)
COVID-19 vaccination status patient	Received any vaccine	51% (409)	56% (111)	47% (94)	70% (140)	32% (64)

Of the adult companions who were not COVID-19 vaccinated and gave reasons for not getting themselves or the PED patient COVID-19 vaccinated, the most common reasons were feeling that the vaccine didn’t work or had too many side effects (Table 3). When looking at the vaccination status of the PED patient and their adult companion, we found that for 49% both were COVID-19 vaccinated, and for 24% neither was vaccinated. It was more common for just the adult companion to be COVID-19 vaccinated (25%) than just the PED patient (3%). The kappa estimate was 0.44 (95% confidence interval 0.38–0.50), significantly different from zero, indicating moderate agreement between COVID-19 vaccination status of the adult companion and PED patient. We excluded five observations from comparison due to missing either the adult companion’s or PED patient’s COVID-19 vaccination status.

**Table 3.** Self-reported barriers to vaccination in unvaccinated by desire to obtain vaccine.

	Reason don’t want vaccine for themselves (N = 189)	Want vaccine for themselves; reason they haven’t gotten it (n = 13)	Reason don’t want vaccine for child patient (n = 318)	Want vaccine for child patient; reason they haven’t gotten it (n = 56)		
Afraid	1% (2)	-	1% (2)		2% (1)
Already had COVID-19	5% (10)	8% (1)	5% (15)		7% (4)
Can’t get an appointment/no appointment at desired location	-	15% (2)	-		13% (7)
Child doesn’t want vaccine	-	-	1% (4)		5% (3)
Don’t think I/child is eligible to get vaccine/not sure when to get it	-	8% (1)	1% (4)		5% (3)
Do not have transportation to vaccine site	-	8% (1)	-		-
Don’t think vaccine works	37% (70)	18% (1)	27% (87)		2% (1)
Underlying health condition	1% (1)	-	1% (3)		7% (4)
Let others get vaccine first	1% (1)	-	3% (9)		-
No reason/unsure	1% (1)	-	1% (2)		-
It is not necessary to be vaccinated	4% (8)	-	4% (13)		-
Other reason: not categorized or blank	2% (4)	-	3% (11)		4% (2)
Other family member doesn’t want child to get vaccine	-	-	2% (5)		7% (4)
People like me/the child don’t get severe COVID-19	3% (5)	-	2% (6)		-
Personal reasons	5% (10)	-	1% (4)		-
Pregnant, planning to get pregnant, or breastfeeding	1% (2)	-	-		-
Religious reasons	3% (5)	-	2% (7)		-
The side effects/risks associated with the vaccine	20% (38)	-	24% (77)		2% (1)
Waiting for more safety data on the vaccine	16% (30)	54% (7)	19% (62)		25% (14)
Waiting to receive physician approval for vaccine	-	-	1% (2)		2% (1)
Work or family commitments/lack of time	1% (2)	-	2% (5)		20% (11)

**Figure 1.**
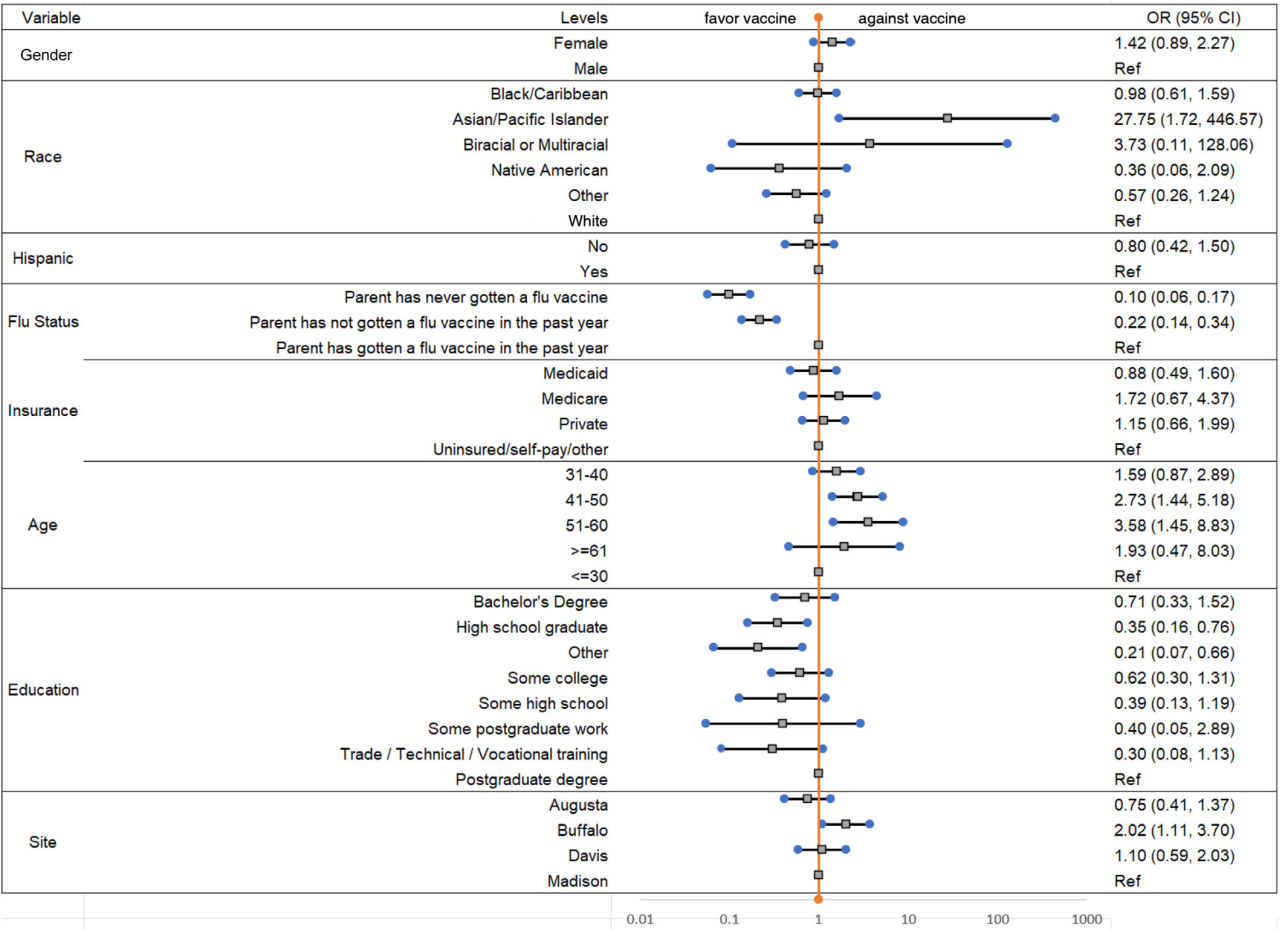
The odds ratios (OR) and 95% confidence intervals (CI) for parent companion vaccinated for different demographic characteristics. Note: Odds ratio for parent companion were estimated using Firth’s penalized likelihood approach.

**Figure 2.**
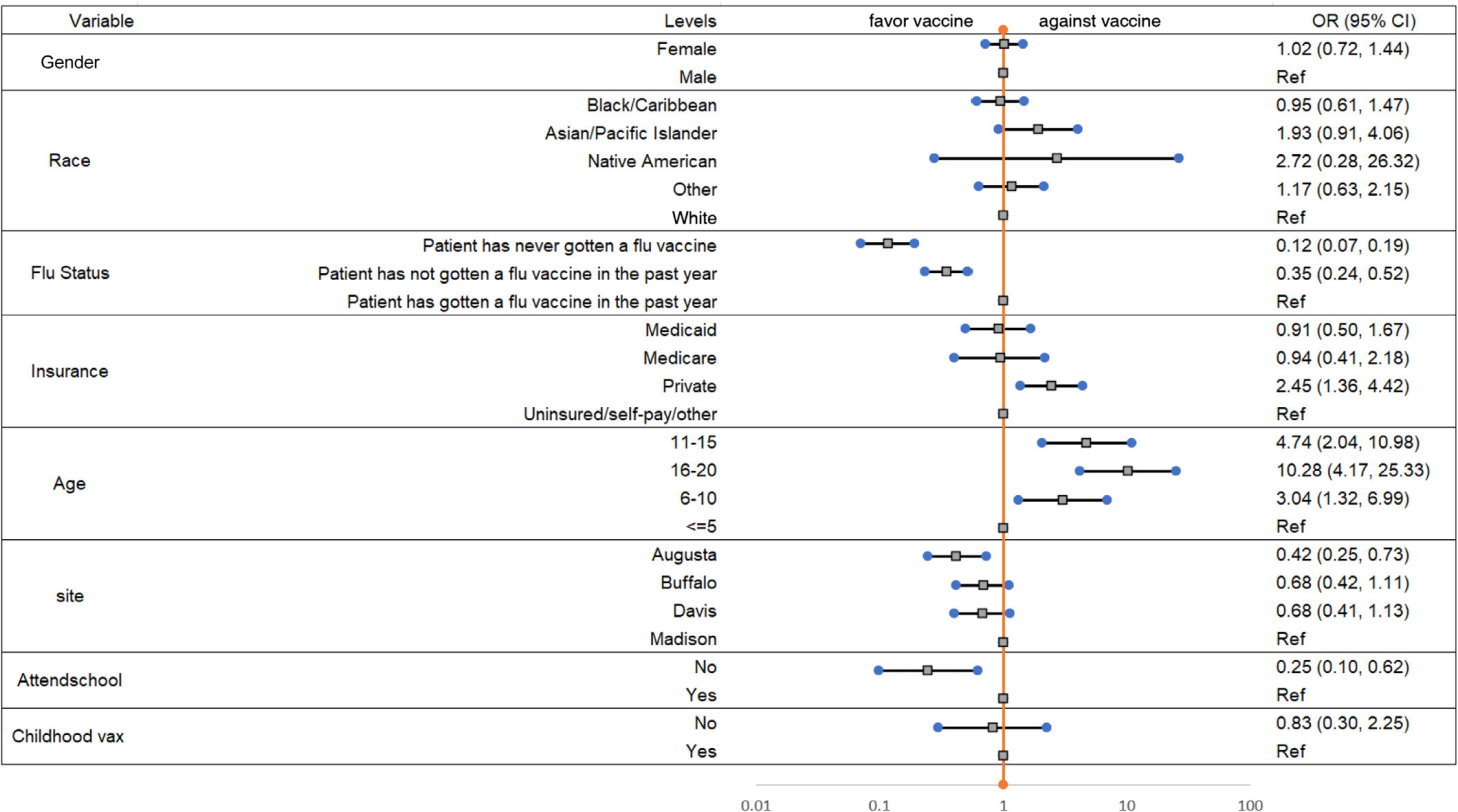
The odds ratios (OR) and 95% confidence intervals (CI) for child patient vaccinated for different demographic characteristics.

Figure 1 shows results of demographic factors associated with adult companion being COVID-19 vaccinated. The odds of being COVID-19 vaccinated for adult companions in Buffalo was 2.02 times that of the Madison site. The results of demographic factors associated with the PED patient being COVID-19 vaccinated is shown in Figure 2. The odds of being COVID-19 vaccinated among PED patients at the Augusta site was 0.42 times that of PED patients at the Madison site. The logistic regression models for adult companions and PED patients were built on 738 and 775 of 800 participants, respectively. The remaining cases were missing data.

## DISCUSSION

In this study we determined that the majority (71.9%) of adult companions of children in the PED had the same COVID-19 vaccination status as the child they were accompanying. Of those with different COVID-19 vaccination status, in most cases (90%) the adult companion had been vaccinated but not the child they were accompanying. This finding may reflect the hesitancy associated with child vaccines, particularly the COVID-19 vaccine.^9^ It may also be the result of vaccine requirements that were placed on adults at this point in the pandemic: for example, the requirement that all federal employees, contractors, international travelers, Head Start education centers, and Centers for Medicare & Medicaid Services-certified facility employees be COVID-19 vaccinated, which was rescinded in 2023.^10^ To our knowledge there were no federal or local requirements at our study sites to vaccinate children, which may have contributed to this finding.

Overall, 49% of the PED patients were not vaccinated. Similar to the results of the survey study of barriers to vaccination of adults presenting to the ED,^5^ a small but significant number (15%) of adult companions of unvaccinated children from across all sites reported wanting the vaccine for the PED patient but had not yet received it. This population should be considered a primary target for increasing the COVID-19 vaccine rate in the pediatric population. Of these cases, one-third identified practical issues as the primary barrier to vaccination of the child. This finding was supported by the World Health Organization (WHO) Strategic Advisory Group of Experts Working on Group Vaccine Hesitancy. They identified practical issues as significant factors when considering vaccination, which included availability, affordability, ease of access, service quality, and respect from health workers.^11^ The other reasons for not vaccinating these children were related to what the WHO report described as “thinking and feeling” issues related to risk and vaccine confidence and “social processes” related to social norms and health worker recommendations. Many of these issues might be able to be addressed through patient/adult companion education that can be provided in the ED. Even if vaccines are not offered in the ED, it may be advantageous to use the ED as a site for providing accurate information on the risks and benefits of COVID-19 vaccination.

## LIMITATIONS

This study may have been limited by the patients who declined participation after being approached. As COVID-19 vaccination can be considered controversial, some individuals may have felt uncomfortable sharing their views and declined participation. It is possible that this group was more likely to not have been vaccinated. Additionally, the survey was read aloud to the patients and their verbal responses were recorded, which may have skewed results as people may have been ashamed to voice their views or fear judgment from medical professionals. Further, we did not study a representative sample of the ED patient population since we would not have approached those with serious or life-threatening illnesses. However, it is likely that any ED vaccination program would focus on this same subset of patients. We also were unable to interview adult companions who did not speak English. Finally, we assumed that the accompanying adult was a primary caregiver of the PED patient. The relationship between the PED patient and the adult companion was not determined or limited to just parents to ensure the study included non-traditional families.

## CONCLUSION

A small but clinically important group of pediatric ED patients were not COVID-19 vaccinated, but their adult companions wanted them to be vaccinated, indicating that consideration should be given to offering vaccination in the ED setting. Most adults accompanying children had the same vaccination status as the pediatric ED patient but, interestingly, for the majority of those with differing vaccine status the adult was vaccinated but not the child. Reasons for avoiding COVID-19 vaccination seemed to center primarily on concerns with efficacy and side effects. Future studies could look at how patient demographics impact vaccination status and the intention to receive vaccination to target education and COVID-19 vaccination initiatives.

## Supplementary Information





## References

[1] Zambrano LD Newhams MM Olson SM et al . BNT162b2 mRNA vaccination against coronavirus disease 2019 is associated with a decreased likelihood of multisystem inflammatory syndrome in children aged 5–18 years-United States, July 2021–April 2022. Clin Infect Dis. 2023;76(3):e90–100. 35924406 10.1093/cid/ciac637 PMC9384630

[2] Vitiello A Ferrara F Troiano V et al . COVID-19 vaccines and decreased transmission of SARS-CoV-2. Inflammopharmacology. 2021;29(5):1357–60. 34279767 10.1007/s10787-021-00847-2 PMC8287551

[3] Centers for Disease Control and Prevention . COVID data tracker. 2024. Available at: https://covid.cdc.gov/covid-data-tracker/#datatracker-home. Accessed July 21, 2023.

[4] Centers for Disease Control and Prevention . Influenza vaccination coverage, children 6 months through 17 years, United States. 2024. Available at: https://www.cdc.gov/flu/fluvaxview/dashboard/vaccination-coverage-race.html. Accessed July 21, 2023.

[5] Harvey BW Kelleran KJ Suffoletto H et al . Emergency department patients’ COVID-19 vaccination status and self-reported barriers. West J Emerg Med. 2022;23(3):292–301. 35679496 10.5811/westjem.2022.1.54615 PMC9183780

[6] Wakefield AJ . MMR vaccination and autism. Lancet. 1999;354(9182):949–50. 10.1016/S0140-6736(05)75696-8 10489978

[7] Baumer-Mouradian SH Kleinschmidt A Servi A et al . Vaccinating in the emergency department, a novel approach to improve influenza vaccination rates via a quality improvement initiative. Pediatr Qual Saf. 2020;5(4):e322. 32766495 10.1097/pq9.0000000000000322 PMC7351463

[8] Baumer-Mouradian SH Servi A Kleinschmidt A et al . Vaccinating in the emergency department, a model to overcome influenza vaccine hesitancy. Pediatr Qual Saf. 2021;6(2):e430. 33855251 10.1097/pq9.0000000000000430 PMC8032353

[9] MacDonald NE , SAGE Working Group on Vaccine Hesitancy. Vaccine hesitancy: definition, scope and determinants. Vaccine. 2015;33(34):4161–4. 25896383 10.1016/j.vaccine.2015.04.036

[10] The White House . The Biden-﻿Harris administration will end COVID-﻿19 vaccination requirements for federal employees, contractors, international travelers, head start educators, and CMS-certified facilities. 2023. Available at: https://www.whitehouse.gov/briefing-room/statements-releases/2023/05/01/the-biden-administration-will-end-covid-19-vaccination-requirements-for-federal-employees-contractors-international-travelers-head-start-educators-and-cms-certified-facilities/. Accessed July 22, 2023.

[11] World Health Organization . Understanding the behavioural and social drivers of vaccine uptake. WHO Position Paper. Wkly Epidemiol Rec. 2022;97(20):209–24.

