# Supplementary material for: Self-Reported COVID-19 Vaccine Status and Barriers for Pediatric Emergency Patients and Caregivers
Source: West J Emerg Med. 2024 Oct 29;26(1):96–102. doi: 10.5811/westjem.18417 (PMC11908513; doi:10.5811/westjem.18417)
Supplement: Supplementary file 1 [file wjem-26-96-s001.docx]

**Appendix 1: Study Survey**

05-02-2023 2:12pm [projectredcap.org](https://projectredcap.org/)

**Completed prior to approaching the participant.**

Triage Category 1

2


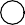

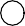

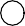

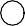

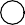


3

4

5

Gender of patient Female

Male Other


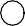

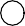

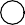


Race of patient African American/African/Black/Caribbean Asian/Pacific Islander

Caucasian Native American Other


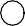

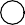

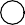

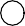

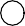


Ethnicity of patient Hispanic or Latino

NOT Hispanic or Latino


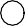

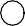


Age of patient

(If the patient is younger than 5 yrs old STOP here)

Chief Complaint

The adult accompanying the patient is able to be Yes

approached for the survey No


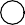

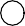


Reason unable to approach the adult accompanying the Infectious precautions

patient Staff states the adult is not able to be


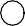

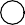


approached at this time (e.g. too distraught) Staff states the patient is too sick


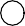

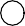

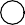

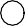

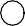


The patient is actively receiving medical care The adult does not speak English

Patient is sleeping Other

**Read After Approaching the participant.**


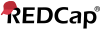


**Introduction:**

**Hello. My name is I am a research associate with the University at Buffalo. Can I talk to**

**you briefly about participating in a research survey that aims to determine the COVID-19 vaccination status of adults who accompany emergency department patients and the patients themselves? The survey should take approximately 15-20 minutes to complete. Participation in the survey requires answering some questions here at the bedside. We will ask some questions about your and the patients' vaccination status.**

**There is a minimal risk to you and the patient of loss of confidentiality from participating in the study, but this is limited since names and other identifying information will not be included in the study data. Only your answers to the questions will be recorded and some general demographic information like your age, race, and zip code. The results of this survey**

**will not necessarily benefit you or the patient directly, but they will** [**help us**](https://projectredcap.org/) **determine how to**

**aim our vaccination efforts. Please note that at this time no COVID-19 vaccines are available here in the emergency department.**

**Dr. Brooke Lerner is the local principal investigator for this study, she can be reached at 716-645-9726 if you have any questions or concerns about the study or you can also contact UB's research participant advocate at 716-888-4845. Your participation is completely voluntary, you can decide to be in the study or not and your decision will not be shared with the clinical staff here in the emergency department and will in no way affect the care of the child you are with. Further, you can stop answering questions at any time. Refusing to participate or not completing the survey will not result in any penalty or loss of benefits to which you are otherwise entitled. Finally, the information collected as part of this study will not be used or distributed for future research studies.**

Would you like to participate in the study? Yes No


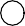

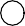

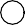


Not approached

How old are you? (Age)

(years)

What gender do you identify as? Female Male

Other


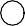

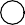

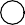


What race(s) do you identify as? (check all that apply)

African American/African/Black/Caribbean Asian/Pacific Islander

Caucasian Native American Other

Prefer Not To Answer

Do you identify as Latinx / Hispanic? Yes No


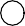

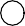


What is the highest level of education you have Some high school

completed? High school graduate


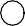

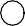

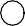

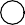

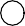

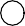

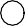

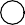


Some college Bachelor's Degree

Trade / Technical / Vocational training Some postgraduate work Postgraduate degree

Other

What type of health insurance do you have? Private Medicare Medicaid

Uninsured / self-pay Other


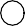

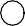

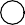

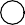

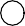

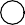

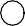


Have you ever been diagnosed with COVID-19? Yes No

Have you received the COVID-19 vaccination? Yes No


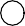

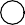


Where did you get vaccinated? Pharmacy

State or County run clinic Healthcare organization clinic Physician's office


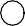

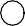

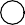

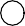

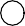


Other

What brand of vaccine did you receive? Pfizer Moderna

Johnson and Johnson Other


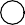

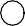

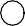

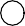

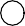

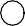


Did you get the recommended second dose? Yes No

If you did not receive a second dose (Pfizer / I have an appointment / too early

Moderna), what prevented you? Don't believe it's necessary Concern regarding side effects


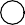

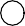

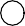

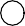

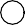


(don't read the choices, listen and select those that Didn't have time

apply or type in other) Appointment was canceled / Couldn't get a second appointment

Waiting due to COVID diagnosis


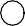

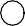


My reaction to the original vaccine was too severe

/ don't want to go through the side effects again
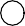
 Other

Did you get a booster? Yes

No


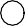

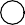


If no booster, what is preventing you from getting a I have an appointment / too early booster? Don't believe it's necessary

Concern regarding side effects (don't read the choices, listen and select those that Didn't have time


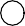

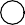

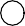

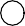

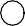


apply or type in other) Appointment was canceled / Couldn't get an appointment

Waiting due to COVID diagnosis


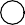

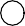


My reaction to the original vaccine was too severe

/ don't want to go through the side effects again
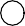
 Other

Please specify

Do you want to get the vaccine? Yes

No


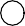

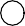


What is keeping you from getting the vaccine? Can't get an appointment

Can't get it at my desired location (e.g. Personal (don't read the choices, listen and select those that physician office)


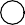

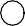


apply or type in other) Don't think I am eligible to get it Waiting for more safety data Already had COVID


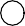

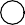

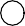

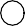

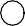

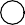


Work or family commitments/time

Do not have transportation to the site Other

Why don't you want to get the vaccine? Cost


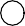

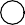

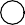

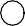

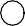

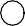

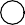

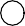

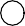

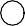

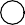


Work or family commitments/time (don't read the choices, listen and select those that Side effects / risks

apply or type in other) Don't think it works

Pregnancy or planning to get pregnant Breastfeeding

Waiting for more safety data Let others get it first Already had COVID

People like me don't get severe COVID Do not have transportation to the site Other

Do you have access to the internet in your home? Yes No

Where have you gotten information about the vaccine? (check as many as apply)

Family / Friends Social media

Primary physician / provider School

Newspaper TV

Radio Podcasts

Personal research Employer (workplace) Religious leaders Other

In the last year did you get vaccinated for the flu? Yes No

Have you ever gotten a flu vaccine? Yes No

**I am now going to ask you questions about the child who is currently a patient here in the emergency department.**

**The following questions are in regards to the patient (Child).**

Does the child attend school? Yes

No

What type of school does the patient attend Traditional

Home / Virtual School

Is your child's health insurance the same as yours? Yes No

If not, what type do they have? Private Medicare Medicaid

Uninsured / self-pay Other

Has the child ever been diagnosed with COVID-19? Yes No

Has the child received the COVID-19 vaccination? Yes No

Where did the child get vaccinated? Pharmacy

State or County run clinic Healthcare organization clinic Physician's office

Other

What brand of vaccine did the child receive? Pfizer Moderna

Johnson and Johnson Other

Did the child get the recommended second dose? Yes No

If the child did not receive a second dose (Pfizer / My Child has an appointment / too early Moderna), what prevented them from getting a second Don't believe it's necessary

dose? Concern regarding side effects

Didn't have time

(don't read the choices, listen and select those that Appointment was canceled / Couldn't get a second apply or type in other) appointment

Waiting due to COVID diagnosis

My Child's reaction to the original vaccine was too severe / don't want to go through the side effects again

Other

Did your child get a booster? Yes

No

If your child did not receive a booster, what My child has an appointment / too early

prevented them from getting a booster? Don't believe it's necessary Concern regarding side effects

(don't read the choices, listen and select those that Didn't have time

apply or type in other) Appointment was canceled / Couldn't get an appointment

Waiting due to COVID diagnosis

My child's reaction to the original vaccine was too severe / don't want to go through the side effects again

Other

Do you want your child to get the COVID-19 vaccine? Yes No

What is keeping your child from getting the vaccine? Can't get an appointment

Can't get it at my desired location (e.g. Personal (don't read the choices, listen and select those that physician's office)

apply or type in other) Don't think my child is eligible to get it Waiting for more safety data

Already had COVID

Work or family commitments/time

Do not have transportation to the site Other

Why don't you want your child to get the COVID-19 Cost

vaccine? Work or family commitments/time

Side effects / risks (don't read the choices, listen and select those that Don't think it works

apply or type in other) Pregnancy or planning to get pregnant Breastfeeding

Waiting for more safety data Let others get it first Already had COVID

Children don't get severe COVID

Do not have transportation to the site Other

Is your child up to date with the required childhood Yes

vaccinations not including the COVID-19 vaccine? No

Did you follow your physician's recommended vaccine Yes schedule or did you ask for modifications? No

I don't know

Has your child's doctor talked to you about getting Yes

them vaccinated against COVID-19? No

If yes, what did they recommend?

In the last year did your child get vaccinated for the Yes

flu? No

Has your child ever received a flu vaccine? Yes No

Do you have other children? Yes

No

Please answer the age and vaccinated status for each child

Age Vaccinated (Yes / No) 2nd Child

3rd Child 4th Child 5th Child 6th Child

Comment(s) or note(s)
